# Moisture chamber goggles for the treatment of postoperative dry eye in patients receiving SMILE and FS-LASIK surgery

**DOI:** 10.1186/s12886-023-03241-4

**Published:** 2023-12-08

**Authors:** Tianze Huang, Yuchen Wang, Zhou Zhu, Qingyang Wu, Di Chen, Ying Li

**Affiliations:** 1 Department of Ophthalmology, Peking Union Medical College Hospital, Chinese Academy of Medical Sciences & Peking Union Medical College, Shuaifuyuan 1, Beijing, 100005 Dongcheng District China; 2 Department of Ophthalmology, the First Affiliated Hospital, School of Medicine, Zhejiang University, Hangzhou, 310003 Zhejiang China

**Keywords:** Dry eye, Small-incision lenticule extraction, Femtosecond laser-assisted in situ keratomileusis, Moisture chamber goggles

## Abstract

**Background:**

The incidence of refractive surgery-related dry eye disease (DED) is rising due to the increasing popularity of corneal refractive surgery. The moisture chamber goggles (MCGs) have been shown to tear evaporation by increasing local humidity and minimizing airflow. The current study aims to evaluate the efficacy of moisture chamber goggles for refractive surgery-related DED.

**Methods:**

In this nonrandomized open-label controlled study, 78 participants (156 eyes) receiving refractive surgery were enrolled between July 2021 and April 2022, and sequentially allocated to MGC and control groups. 39 participants were allocated to the MGC groups, of which 53.8% received small-incision lenticule extraction (SMILE) and 46.2% received femtosecond laser-assisted in situ keratomileusis (FS-LASIK), and were instructed to wear MCGs for the duration of 1 month postoperatively, in addition to the standard postoperative treatment received by the control groups (56.4% SMILE, 43.6% FS-LASIK). Participants underwent full ophthalmic examinations, including visual acuity, manifest refraction, DED evaluations, and higher-order aberrations (HOAs), both preoperatively and at routine follow-ups 1 day, 1 week, and 1 month after surgery. DED parameters included non-invasive tear film break-up time (NIBUT), tear meniscus height (TMH), conjunctival congestion, lipid layer thickness (LLT), and ocular surface disease index (OSDI) questionnaires. Student’s t-test was used for comparisons between control and MCG groups, and between preoperative and postoperative parameters within groups.

**Results:**

Postoperative NIBUT decreased in both SMILE and FS-LASIK control groups 1 day after the surgery (SMILE, *P* = 0.001; FS-LASIK, *P* = 0.008), but not in the corresponding MCG groups (SMILE, *P* = 0.097; FS-LASIK, *P* = 0.331). TMH in the MCG group was significantly higher at 1 week (*P* = 0.039) and 1 month (*P* = 0.015) in SMILE, and 1 day (*P* = 0.003) in FS-LASIK groups. In FS-LASIK participants, significantly lower HOAs and coma levels in the MCG group were observed 1 day (total HOAs, *P* = 0.023; coma, *P* = 0.004) and 1 week (total HOAs, *P* = 0.010, coma, *P* = 0.004) after surgery. No consistent statistically significant intergroup difference was observed between MCG and control groups in conjunctival congestion, LLT, and OSDI.

**Conclusions:**

MCGs effectively slowed tear evaporation, increased tear film stability, and improved HOAs in patients receiving SMILE and FS-LASIK surgeries. MCG is an effective adjuvant therapy in the comprehensive management of refractive surgery-related DED.

## Background

Dry eye disease (DED) is a multifactorial disease of the ocular surface, characterized by the loss of homeostasis of the tear film. DED can be induced by a variety of iatrogenic factors, including corneal refractive surgery [1, 2]. The increasing popularity of refractive surgery is accompanied by the increasing prevalence of refractive surgery-related DED. Previous studies have demonstrated that refractive surgery inevitably leads to transient ocular surface disturbances postoperatively, with varying incidence and severity between studies [3–6]. A number of etiologies have been shown to contribute to refractive surgery-related DED, including prior history of DED, meibomian gland function, ocular inflammation, choice of surgical techniques, and damage to the corneal subbasal nerve plexuses [7, 8].

The current consensus recommended comprehensive management for surgically-induced DED, encompassing both preoperative and postoperative approaches [9, 10]. The Tear Film and Ocular Surface (TFOS) Dry Eye Workshop (DEWS) II reports stressed the importance of treatment of preexisting DED before surgery, postoperative tear replacement, tear conservation, and anti-inflammation therapies [1, 9]. Moisture chamber goggles (MCGs) were recommended as a basic approach for tear conservation. They have been shown to slow tear evaporation by increasing local humidity and minimizing airflow. Previous studies have focused on the short-term efficacy of MCGs in DED patients, as well as in patients exposed to adverse environment [11–14]. However, the long-term efficacy of MCGs and their effects on refractive surgery recipients require further investigation.

This prospective controlled intervention study aims to explore the effects of MCG on refractive surgery-related DED, by comparing the visual outcomes and DED parameters between the control and MCG-treated participants in the postoperative period.

## Patients and methods

### Study design

This nonrandomized open-label controlled study enrolled patients who underwent small-incision lenticule extraction (SMILE) and femtosecond laser-assisted in situ keratomileusis (FS-LASIK) procedures between July 2021 and April 2022 at the Department of Ophthalmology, Peking Union Medical College Hospital (PUMCH). The participants were sequentially allocated to MCG and control groups. The study adhered to the tenets of the Declaration of Helsinki and was supervised by the institutional review board at PUMCH. Signed informed consent was obtained from all patients.

Inclusion criteria were as follows: (1) age between 18 and 40 years; (2) stable refractive error (≤ 0.5 D change of refractive error) in the past 1 year; (3) spherical equivalent (SE) between -2.50 D and -20.00 D; (4) astigmatism up to -5.00 D; (5) logarithm of the minimal angle of resolution (LogMAR) of BCVA of 0.1 or better; (6) clear crystalline lens.

Exclusion criteria included current or history of severe ophthalmic diseases including corneal diseases, cataracts, glaucoma, retinal detachment, neuro-ophthalmic diseases, trauma, ocular surgery, and diagnosed autoimmune diseases. Patients with severe preoperative dry eye, defined as prominent desiccation before surgery, a fluorescein TBUT < 2 s, and/or corneal epithelial defects in 2 quadrants or more and/or fluorescein staining ≥ 30, were excluded from the study [15, 16].

All participants underwent complete preoperative ophthalmic examinations, including uncorrected visual acuity (UCVA), best corrected visual acuity (BCVA), manifest and cycloplegic refraction by autorefractometery (RM-800, Topcon, Japan), standard slit-lamp biomicroscopy and funduscopic examinations, gonioscopy, intraocular pressure (IOP) by a non-contact tonometer (Canon, Japan), and corneal topography (Canon, Japan). The participants underwent routine follow-ups at 1 day, 1 week, and 1 month postoperatively, with examinations including BCVA, manifest refraction, standard slit-lamp biomicroscopy, and IOP measurement. OSDI, DED evaluation, and HOA examinations were performed at the preoperative examination and each follow-up session. Participants unable to complete preoperative examinations or more than 1 follow-up examinations were excluded from the analyses.

### Surgical procedures

Surgical procedures were performed by an experienced surgeon (YL). SMILE was performed under topical anesthesia, with the VisuMax 500 kHz femtosecond laser (Carl Zeiss Meditec, Germany). Cap thickness was set at 110–120 μm, cap diameter at 7.0–7.5 mm, and lenticules diameter at 6.0–6.5 mm, with a transition zone of 0.1 mm. A 2 mm side cut incision was made at the 10 o’clock position of the cornea. Cut energy was set at 135 nJ. The stromal lenticules were removed using forceps.

FS-LASIK was performed using the VisuMax 500 kHz femtosecond laser (Carl Zeiss Meditec, Germany) for flap creation, and the Schwind Amaris 179 excimer laser (Schwind Eye-Tech-Solutions, Germany) for refractive correction. Flap thickness was set at 90 or 100 μm, flap diameter at 8.5 mm, and hinged at the 12 o’clock position of the cornea. The side cut angle was at 120 degrees.

The prescriptions for postoperative management were as follows: tobramycin dexamethasone eye drops (s.a. Alcon-Couvreur n.v.), four times a day for two weeks, deproteinized calf blood extract eye gel (Xingqi pharmaceuticals), once for four weeks, and sodium hyaluronate eye drops, four times a day for four weeks.

### Moisture chamber goggle

The moisture chamber goggle consists of two moisture-retaining chambers and supporting structures. The chambers have rubber adapters to better fit the frame of the user, providing better sealing and a more comfortable wearing experience. At each junction of the chamber and the temples, a tank connected to the chamber, filled with water-absorbing material, was designed to provide moisture and maintain humidity levels in the chambers. The participants were asked to fill the tanks each time before use. The participants were instructed to use MCG for at least half of the day according to instruction postoperatively for 1 month.

### DED evaluation

DED-1L Dry Eye Analyzer (Kanghuaruiming Science Technology, China) is a comprehensive dry eye diagnostic system that performs non-invasive tear film break-up time (NIBUT), tear meniscus height (TMH), lipid layer interferometry, and congestion assessment [17]. NIBUT and TMH measurement was performed under infrared light, while lipid layer interferometry and congestion examination was conducted under natural white illumination. NIBUT is automatically measured as the duration from the last complete blink to the first discontinuity in Placido ring reflections under infrared illumination. TMH was calculated as the average of three measurements at central, medial, and lateral paracentral locations. The congestion levels in the conjunctival and perilimbal regions were automatically measured under natural light, and an average congestion score was calculated.

The LipiView Ocular Surface Interferometer (TearScience, United States) measures the absolute thickness of the lipid layer using interferometric images of the tear film. During the examination, the participants were asked to maintain still and blink normally. The camera was adjusted to focus on the tear film plane and captured a 25-s video with a clear interferometric image of the tear film [18]. Lipid layer thickness (LLT) is measured in interferometric color units (ICU). LipiView automatically calculates the average (AvgICU), maximal (MaxICU), and minimal (MinICU) measurements of LLT. The homogeneity in lipid layer distribution is reflected by $DevICU= \sqrt{{(MaxICU-AvgICU)}^{2}+{(MinICU-AvgICU)}^{2}}$.

To avoid introducing bias from diurnal variations, objective DED evalutions were performed in the mornings. The participants were asked to complete the OSDI questionnaire at preoperative examination and 1-month follow-up.

### HOA evaluation

The iTrace aberrometer (Tracey Technologies, United States) was used to measure ocular HOAs following 10 min of dark adaptation without pharmacological pupil dilatation before the operation and at the follow-ups. The root means square values of total HOAs, spherical aberration, secondary astigmatism, coma aberration, and trefoil aberration were recorded.

### Statistical analysis

Statistical analyses were performed with R (version 4.2.3) and RStudio (2023.03.1 + 446). BCVA and UCVA were converted to logMAR visual acuity. Continuous variables were presented as mean ± standard deviation (SD) under normal distribution or median with interquartile range (IQR) under non-normal distribution. Independent student’s t-test was used for comparisons of parameters between control and MCG groups. Paired student’s t-test was used for comparisons between preoperative and postoperative parameters within a group. A p-value of less than 0.05 was considered statistically significant.

## Results

### Demographics and clinical characteristics

Demographics and preoperative parameters are shown in Table 1. The SMILE control and MCG groups included 22 (27.3% male, 72.7% female) and 21 (19.0% male, 81.0% female) participants, and The FS-LASIK control and MCG groups included 17 (23.5% male, 76.5% female) and 18 (16.7% male, 83.3% female) participants, respectively. The mean age was 28.41 ± 6.08 and 26.67 ± 4.48 years for SMILE control and MCG groups (*P* = 0.290), and 30.41 ± 5.82 and 31.61 ± 10.58 years for FS-LASIK control and MCG groups (*P* = 0.680), respectively. There was no statistically significant difference in preoperative SE, visual acuity, total HOA, and main DED parameters.

**Table 1:** Demographics and preoperative parameters

	SMILE			FS-LASIK		
	Control, n = 22	MCG, n = 21	*P*	Control, n = 17	MCG, n = 18	*P*
Age (years old)	28.41 ± 6.08	26.67 ± 4.48	0.290	30.41 ± 5.82	31.61 ± 10.58	0.680
M/F	6/16	4/17	0.721^a^	4/13	3/15	0.691^a^
Preop MRSE(D)	-5.27 ± 1.48	-5.43 ± 1.45	0.611	-8.10 ± 1.81	-7.31 ± 1.52	0.053
BCVA (logMAR)	0.004 ± 0.020	0.002 ± 0.015	0.587	-0.003 ± 0.048	-0.012 ± 0.043	0.377
IOP (mmHg)	15.8 ± 2.95	16.3 ± 2.95	0.484	15.8 ± 2.65	15.3 ± 2.45	0.387
NIBUT(s)	9.27 ± 5.90	9.60 ± 7.22	0.819	10.16 ± 6.96	8.78 ± 6.40	0.389
TMH(mm)	0.24 ± 0.07	0.24 ± 0.05	0.819	0.21 ± 0.06	0.22 ± 0.06	0.426
AvgICU (nm)	62.5 ± 28.1	67.0 ± 24.7	0.428	76.4 ± 24.8	73.6 ± 23.2	0.798
Total HOA	0.490 ± 0.292	0.407 ± 0.221	0.142	0.728 ± 0.676	0.608 ± 0.409	0.272
OSDI	24.0 ± 21.0	19.0 ± 7.2	0.432	17.7 ± 6.3	18.5 ± 10.7	0.877

Independent student’s t-test was used for comparisons between control and MGC groups

*SMILE* small-incision lenticule extraction, *FS-LASIK* femtosecond laser-assisted in situ keratomileusis, *MCG* moisture chamber goggles, *MRSE* preoperative manifest refractive spherical equivalent, *BCVA* best-corrected visual acuity, *IOP* intraocular pressure, *HOA* high-order aberration, *NIBUT* non-invasive tear film break-up time, *TMH* tear meniscus height, *AvgICU* average lipid layer thickness in interference color unit, *HOA* higher-order aberrations, *OSDI* ocular surface disease index

^a^Fisher’s exact test

### Visual acuity and postoperative SE

**Fig. 1:**
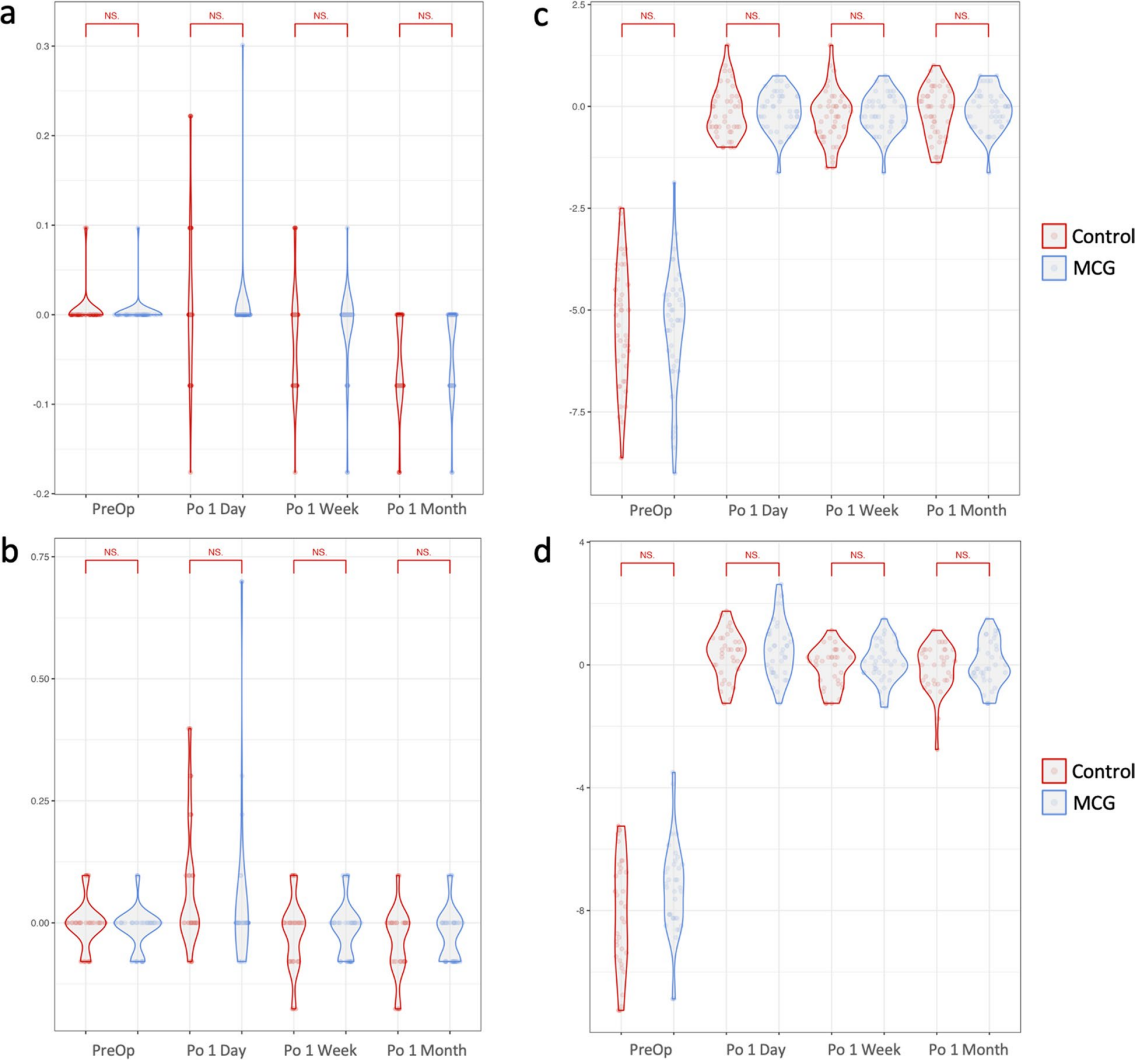
Visual acuity and manifest refractive spherical equivalent after moisture chamber goggles treatment at postoperative 1 day, 1 week, and 1 month. **A** Visual acuity of control and MCG groups in SMILE participants, best-corrected visual acuity (BCVA) in log MAR preoperatively, and uncorrected visual acuity (UCVA) postoperatively; **B** Visual acuity of control and MCG groups in FS-LASIK participants, BCVA preoperatively and UCVA postoperatively; **C** MRSE of control and MCG groups in SMILE participants; **D** MRSE of control and MCG groups in FS-LASIK participants. Independent student’s t-test for comparisons between control and MGC groups. MRSE, manifest refractive spherical equivalent; MCG, moisture chamber goggles; SMILE, small-incision lenticule extraction; FS-LASIK, femtosecond laser-assisted in situ keratomileusis; PreOp, preoperative; Po, postoperative; NS, not significant

In both SMILE control and MCG groups, postoperative UCVA significantly improved compared to preoperative BCVA at 1 week (control, *P* = 0.002; MCG, *P* = 0.024) and 1 month (control, *P* < 0.001; MCG, *P* < 0.001) after surgery, but not at 1 day (control, *P* = 0.322; MCG, *P* = 0.525) (Tables 2 and 3). In FS-LASIK groups, postoperative UCVA was significantly better at 1 day in both control (*P* = 0.001) and MCG groups (*P* = 0.030), and 1 month in the control group (*P* = 0.004), but not at 1 week (control, *P* = 0.179; MCG, *P* = 0.923) or 1 month in the MCG group (*P* = 0.268). Postoperative MRSE significantly improved compared to preoperative at 1 day, 1 week, and 1 month after surgery for both SMILE and FS-LASIK control and MCG groups (*P* < 0.001). No statistically significant intergroup difference was observed in postoperative UCVA and MRSE in both SMILE and FS-LASIK groups (Fig. 1).

**Table 2:** Postoperative parameters 1 day, 1 week, and 1 month after SMILE surgery

	Control							MCG						
	Preop	PO 1 Day	PO 1 Week	PO 1 Month	*P*_*1*_	*P*_*2*_	*P*_*3*_	PreOp	PO 1 Day	PO 1 Week	PO 1 Month	*P*_*1*_	*P*_*2*_	*P*_*3*_
UCVA^a^	0.004 ± 0.020	0.018 ± 0.092	-0.024 ± 0.061	-0.052 ± 0.051	0.322	0.002^**^	< 0.001^***^	0.002 ± 0.015	0.007 ± 0.046	-0.016 ± 0.048	-0.037 ± 0.049	0.525	0.024^*^	< 0.001^**v^
MRSE(D)	-5.27 ± 1.48	-0.13 ± 0.61	-0.25 ± 0.64	-0.14 ± 0.61	< 0.001^***^	< 0.001^***^	< 0.001^***^	-5.43 ± 1.45	-0.11 ± 0.49	-0.16 ± 0.47	-0.10 0.49	< 0.001^***^	< 0.001^***^	< 0.001^***^
NIBUT(s)	9.27 ± 5.90	5.49 ± 4.61	8.48 ± 6.96	8.20 ± 5.29	0.001^**^	0.543	0.269	9.60 ± 7.22	7.29 ± 6.71	11.90 ± 9.25	10.76 ± 6.91	0.097	0.176	0.445
TMH(mm)	0.24 ± 0.07	0.22 ± 0.06	0.21 ± 0.05	0.21 ± 0.06	0.070	0.011^*^	0.003^**^	0.24 ± 0.05	0.23 ± 0.07	0.24 ± 0.07	0.24 ± 0.05	0.285	0.947	0.775
Congestion	1.124 ± 0.199	1.103 ± 0.202	1.128 ± 0.208	1.108 ± 0.160	0.531	0.830	0.539	1.187 ± 0.246	1.101 ± 0.267	1.057 ± 0.214	1.056 ± 0.218	0.061	0.002^**^	0.004^**^
AvgICU	62.48 ± 28.15	62.41 ± 27.67	52.50 ± 24.65	57.05 ± 24.40	0.990	0.037^*^	0.290	67.02 ± 24.72	56.29 ± 19.85	49.55 ± 20.87	55.45 ± 25.08	0.023^*^	0.001^**^	0.014^*^
DevICU	21.82 ± 16.75	16.33 ± 17.35	16.84 ± 12.98	18.27 ± 12.61	0.176	0.117	0.247	17.28 ± 12.44	17.07 ± 12.66	19.68 ± 13.02	19.89 ± 12.45	0.940	0.402	0.305
Total HOA	0.490 ± 0.292	0.243 ± 0.164	0.237 ± 0.145	0.244 ± 0.144	< 0.001^***^	< 0.001^***^	< 0.001^***^	0.407 ± 0.221	0.252 ± 0.152	0.262 ± 0.229	0.253 ± 0.205	< 0.001^***^	0.004^**^	0.001^**^
Coma	0.276 ± 0.198	0.137 ± 0.121	0.159 ± 0.120	0.168 ± 0.143	< 0.001^***^	0.002^**^	0.003^**^	0.241 ± 0.187	0.146 ± 0.108	0.167 ± 0.172	0.160 ± 0.155	0.006^**^	0.058	0.032^*^
Spherical	0.141 ± 0.178	0.004 ± 0.105	-0.019 ± 0.084	0.008 ± 0.090	< 0.001^***^	< 0.001^***^	< 0.001^***^	0.062 ± 0.151	-0.018 ± 0.138	0.002 ± 0.156	0.031 ± 0.129	0.002^**^	0.051	0.285
SA	0.088 ± 0.076	0.052 ± 0.046	0.047 ± 0.033	0.049 ± 0.046	0.009^**^	0.001^**^	0.002^**^	0.093 ± 0.068	0.052 ± 0.042	0.054 ± 0.045	0.056 ± 0.050	0.002^**^	0.001^**^	0.004^**^
Trefoil	0.225 ± 0.176	0.123 ± 0.099	0.111 ± 0.098	0.086 ± 0.053	0.001^**^	< 0.001^***^	< 0.001^***^	0.177 ± 0.105	0.110 ± 0.070	0.114 ± 0.071	0.105 ± 0.083	0.001^**^	0.003^**^	0.001^**^
OSDI	24.0 ± 21.0			30.1 ± 20.0			0.265	19.0 ± 7.2			20.3 ± 8.9			0.663

*P*_*1*_, PreOp vs. Po 1 day; *P*_*2*_, PreOp vs. Po 1 week; *P*_*3*_, PreOp vs. Po 1 month

*MCG* moisture chamber goggles, *PreOp* preoperative, *Po* postoperative, *UCVA* uncorrected visual acuity, *MRSE* manifest refractive spherical equivalent, *TMH* tear meniscus height, *NIBUT* non-invasive tear film break-up time, *AvgICU* average lipid layer thickness in interferometric color units, *DevICU* deviation of lipid layer thickness in interferometric color units, *HOA* higher-order aberrations, *SA* secondary astigmatism, *OSDI* ocular surface disease index

^*^p < 0.05, **p < 0.01, ***p < 0.001, paired student’s t-test

^a^Best-corrected visual acuity (BCVA) for preoperative examination

**Table 3:** Postoperative parameters 1 day, 1 week, and 1 month after FS-LASIK surgery

	Control							MCG						
	PreOp	Po 1 Day	Po 1 Week	Po 1 Month	*P*_*1*_	*P*_*2*_	*P*_*3*_	PreOp	Po 1 Day	Po 1 Week	Po 1 Month	*P*_*1*_	*P*_*2*_	*P*_*3*_
UCVA^a^	-0.003 ± 0.048	0.074 ± 0.129	-0.022 ± 0.074	-0.041 ± 0.069	0.001^**^	0.179	0.004^**^	-0.012 ± 0.043	0.052 ± 0.174	-0.013 ± 0.053	-0.013 ± 0.053	0.030^*^	0.923	0.268
MRSE(D)	-8.10 ± 1.81	0.32 ± 0.76	-0.07 ± 0.65	-0.12 ± 0.77	< 0.001^***^	< 0.001^***^	< 0.001^***^	-7.31 ± 1.52	0.50 ± 0.90	0.11 ± 0.65	0.02 ± 0.73	< 0.001^***^	< 0.001^***^	< 0.001^***^
NIBUT(s)	10.17 ± 6.96	6.20 ± 5.29	6.91 ± 6.72	6.81 ± 4.95	0.008^**^	0.037^*^	0.033^*^	8.78 ± 6.40	7.27 ± 7.03	8.25 ± 6.63	9.04 ± 6.40	0.331	0.749	0.857
TMH(mm)	0.21 ± 0.06	0.20 ± 0.06	0.20 ± 0.04	0.22 ± 0.07	0.383	0.333	0.557	0.22 ± 0.06	0.26 ± 0.09	0.22 ± 0.06	0.22 ± 0.05	0.024^*^	0.887	0.628
Congestion	1.234 ± 0.159	1.164 ± 0.223	1.255 ± 0.206	1.125 ± 0.116	0.144	0.562	0.001^**^	1.158 ± 0.184	1.217 ± 0.365	1.055 ± 0.146	1.198 ± 0.237	0.289	< 0.001^***^	0.221
AvgICU	76.35 ± 24.82	81.71 ± 23.10	72.94 ± 26.87	80.76 ± 20.74	0.381	0.597	0.360	73.61 ± 23.24	77.33 ± 23.98	59.44 ± 18.23	69.94 ± 25.80	0.456	0.004^**^	0.442
DevICU	18.52 ± 16.97	15.31 ± 13.86	21.53 ± 18.66	24.72 ± 18.79	0.356	0.441	0.161	20.49 ± 14.14	11.89 ± 12.28	20.14 ± 11.65	17.86 ± 15.14	0.005^**^	0.894	0.438
Total HOA	0.728 ± 0.676	0.414 ± 0.285	0.355 ± 0.266	0.320 ± 0.235	0.025^*^	0.002^**^	0.001^**^	0.608 ± 0.409	0.282 ± 0.167	0.221 ± 0.132	0.280 ± 0.177	< 0.001^***^	< 0.001^***^	< 0.001^***^
Coma	0.317 ± 0.350	0.266 ± 0.232	0.266 ± 0.233	0.260 ± 0.207	0.538	0.442	0.349	0.303 ± 0.277	0.138 ± 0.085	0.133 ± 0.100	0.193 ± 0.160	0.002^**^	0.002^**^	0.058
Spherical	0.138 ± 0.240	-0.010 ± 0.137	0.068 ± 0.170	0.064 ± 0.141	0.002^**^	0.091	0.113	0.125 ± 0.165	-0.006 ± 0.097	0.021 ± 0.075	0.037 ± 0.094	< 0.001^***^	0.001^**^	0.005^**^
SA	0.155 ± 0.154	0.087 ± 0.071	0.061 ± 0.051	0.031 ± 0.027	0.030^*^	< 0.001^***^	< 0.001^***^	0.128 ± 0.118	0.077 ± 0.058	0.047 ± 0.046	0.066 ± 0.050	0.031	0.001^**^	0.004^**^
Trefoil	0.403 ± 0.414	0.163 ± 0.152	0.100 ± 0.064	0.092 ± 0.055	0.002^**^	< 0.001^***^	< 0.001^***^	0.296 ± 0.211	0.121 ± 0.103	0.118 ± 0.135	0.113 ± 0.077	< 0.001^***^	< 0.001^***^	< 0.001^***^
OSDI	17.7 ± 6.3			31.8 ± 12.0			0.020^*^	18.5 ± 10.7			14.7 ± 12.3			0.200

*P*_*1*_, PreOp vs. Po 1 day; *P*_*2*_, PreOp vs. Po 1 week; *P*_*3*_, PreOp vs. Po 1 month

*MCG* moisture chamber goggles, *PreOp* preoperative, *Po* postoperative, *UCVA* uncorrected visual acuity, *MRSE* manifest refractive spherical equivalent, *TMH* tear meniscus height, *NIBUT* non-invasive tear film break-up time, *AvgICU* average lipid layer thickness in interferometric color units, *DevICU* deviation of lipid layer thickness in interferometric color units, *HOA* higher-order aberrations, *SA* secondary astigmatism, *OSDI* ocular surface disease index

*p < 0.05, **p < 0.01, ***p < 0.001, paired student’s t-test

^a^Best-corrected visual acuity (BCVA) for preoperative examination

### Dry eye parameters

#### Non-invasive tear break-up time

**Fig. 2:**
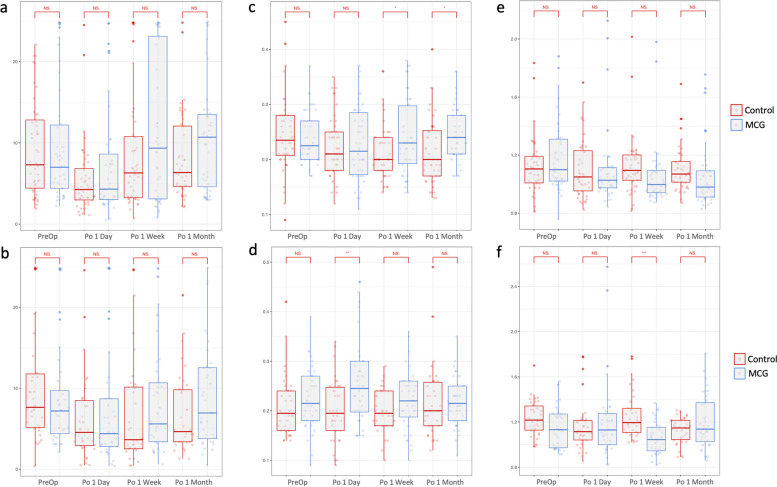
Tear meniscus height, non-invasive tear film break-up time, and congestion evaluation after moisture chamber goggles treatment at postoperative 1 day, 1 week, and 1 month. **A** TMH of control and MCG groups in SMILE participants; **B** TMH of control and MCG groups in FS-LASIK participants; **C** NIBUT of control and MCG groups in SMILE participants; **D** NIBUT of control and MCG groups in FS-LASIK participants; **E** Congestion score of control and MCG groups in SMILE participants; **F** Congestion score of control and MCG groups in FS-LASIK participants. *p < 0.05, **p < 0.01, ***p < 0.001, independent student’s t-test. TMH, tear meniscus height; NIBUT, non-invasive tear film break-up time; MCG, moisture chamber goggles; SMILE, small-incision lenticule extraction; FS-LASIK, femtosecond laser-assisted in situ keratomileusis; PreOp, preoperative; Po, postoperative; NS, not significant

In both SMILE and FS-LASIK control groups, postoperative NIBUT decreased 1 day after the surgery (SMILE, *P* = 0.001; FS-LASIK, *P* = 0.008), but not in the corresponding MCG groups (SMILE, *P* = 0.097; FS-LASIK, *P* = 0.331) (Tables 2 and 3). This effect persisted in the FS-LASIK control group at 1 week (*P* = 0.037) and 1 month (*P* = 0.033) postoperatively, but not in the SMILE control group. Intergroup differences between control and MCG groups were not statistically significant at each follow-up in both SMILE and FS-LASIK participants (Fig. 2).

#### Tear meniscus height

A significant decrease in TMH was observed in SMILE control groups at 1 week (*P* = 0.011) and 1 month (*P* = 0.003) follow-ups, and a significant increase in FS-LASIK MCG groups 1 day postoperatively (*P* = 0.024) (Tables 2 and 3). In both SMILE and FS-LASIK participants, TMH measurements in the MCG groups were higher than those in the control groups (Fig. 2). The comparison was statistically significant at 1 week (*P* = 0.039) and 1 month (*P* = 0.015) postoperatively in the SMILE groups, and 1 day (*P* = 0.003) in the FS-LASIK groups.

#### Conjunctival congestion

The conjunctival congestion level was generally lower in the MCG groups compared to the control groups (Tables 2 and 3). A significant decrease in postoperative congestion score was observed in the SMILE MCG group at 1 week (*P* = 0.002) and 1 month (*P* = 0.004) after surgery, FS-LASIK control group at 1 month (*P* = 0.001), and FS-LASIK MCG group at 1 week (*P* < 0.001). No statistically significant intergroup difference existed except in FS-LASIK groups at 1-week follow-up (*P* < 0.001) (Fig. 2).

#### Lipid layer thickness

**Fig. 3:**
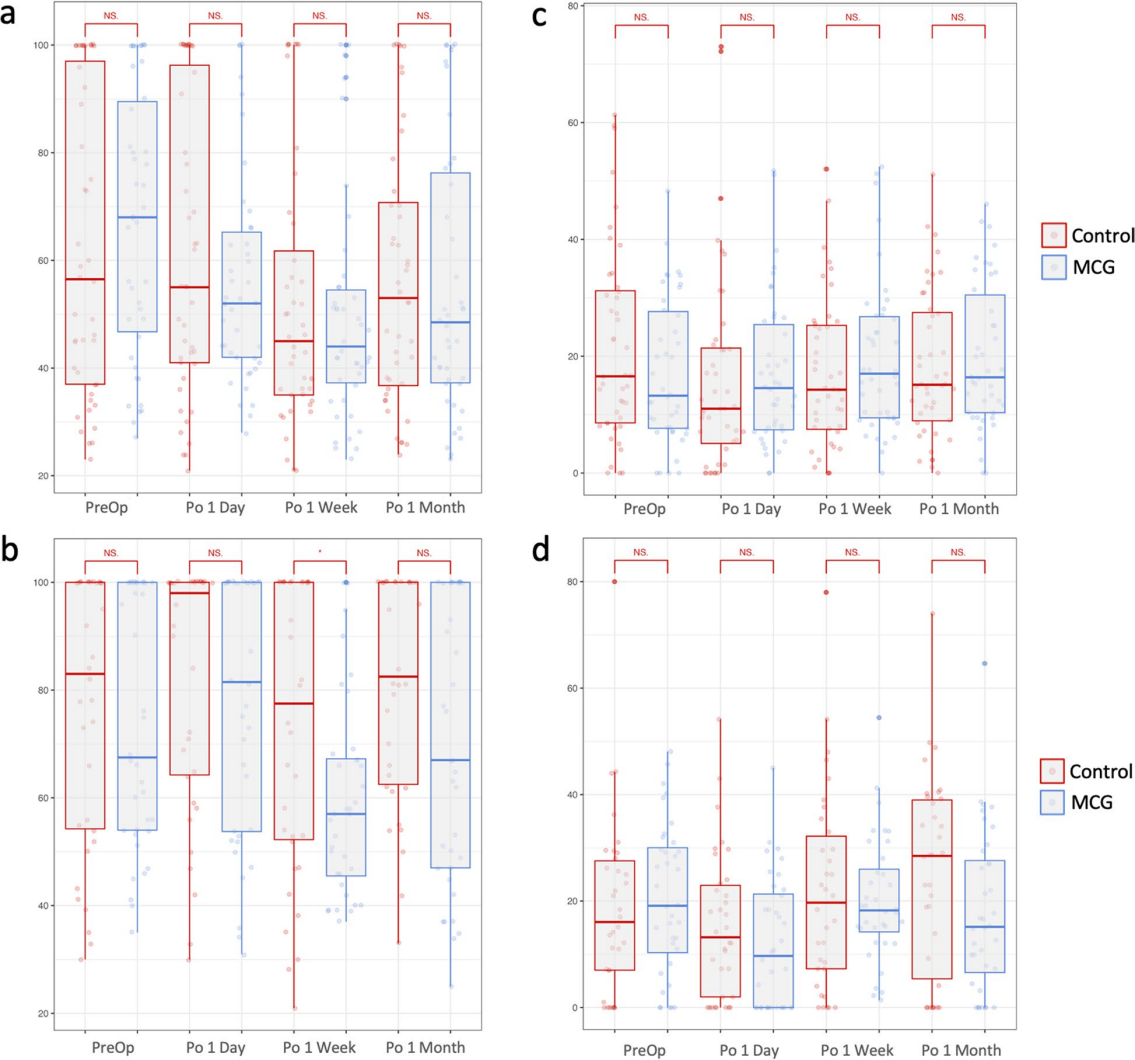
Lipid layer assessment after moisture chamber goggles treatment at postoperative 1 day, 1 week, and 1 month. **A** AvgICU of control and MCG groups in SMILE participants; **B** AvgICU of control and MCG groups in FS-LASIK participants; **C** DevICU of control and MCG groups in SMILE participants; **D** DevICU of control and MCG groups in FS-LASIK participants. *p < 0.05, independent student’s t-test. AvgICU, average lipid layer thickness in interferometric color units; DevICU, deviation in lipid layer thickness in interferometric color units; MCG, moisture chamber goggles; SMILE, small-incision lenticule extraction; FS-LASIK, femtosecond laser-assisted in situ keratomileusis; PreOp, preoperative; Po, postoperative; NS, not significant

A significant decrease in AvgICU was observed in the SMILE MCG group in all three follow-ups (1 day, *P* = 0.023; 1 week, *P* = 0.001; 1 month, *P* = 0.014), and the SMILE control group (*P* = 0.037) and FS-LASIK MCG group (*P* = 0.004) at 1 week postoperatively. No significant intergroup difference was observed in AvgICU except between FS-LASIK groups 1 week after the surgery (*P* = 0.018) (Fig. 3). DevICU was significantly decreased in the FS-LASIK MCG group 1 day postoperatively (*P* = 0.005). DevICU showed no significant intergroup difference.

### Higher-order aberrations

**Fig. 4:**
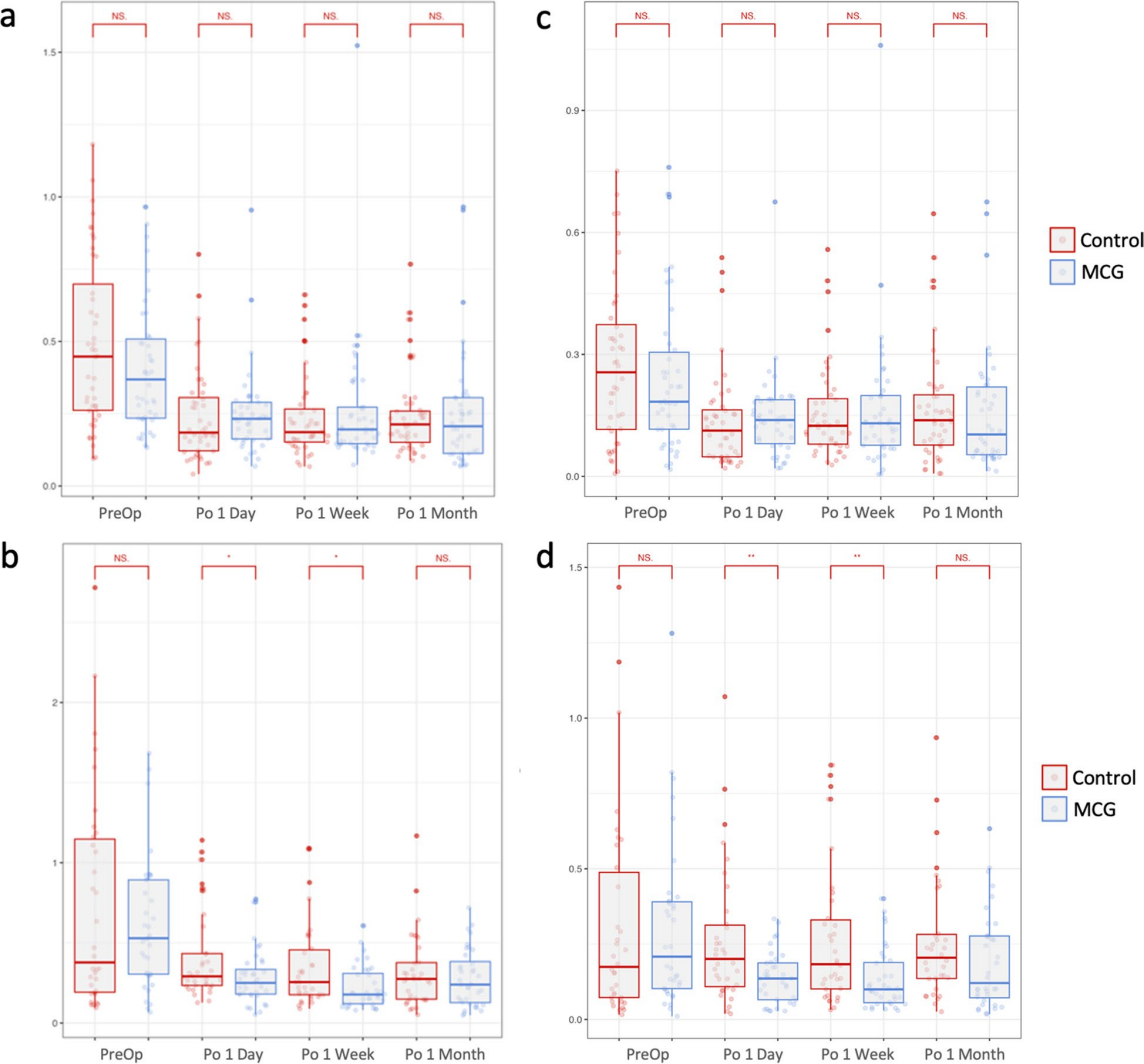
Higher-order aberrations evaluation after moisture chamber goggles treatment at postoperative 1 day, 1 week, and 1 month. **A** Total HOA of control and MCG groups in SMILE participants; **B** Total HOA of control and MCG groups in FS-LASIK participants; **C** Coma of control and MCG groups in SMILE participants; **D** Coma of control and MCG groups in FS-LASIK participants. *p < 0.05, **p < 0.01, independent student’s t-test. HOA, higher-order aberrations; MCG, moisture chamber goggles; SMILE, small-incision lenticule extraction; FS-LASIK, femtosecond laser-assisted in situ keratomileusis; PreOp, preoperative; Po, postoperative; NS, not significant

Postoperative HOA significant decrease compared to preoperative in both SMILE and FS-LASIK groups (Tables 2 and 3). In FS-LASIK participants, the MCG group had significantly lower total HOAs, as well as coma levels 1 day (total HOAs, *P* = 0.023; coma, *P* = 0.004) and 1 week (total HOAs, *P* = 0.010, coma, *P* = 0.004) after surgery compared to the control group (Fig. 4). No significant intergroup difference was observed in SMILE participants postoperatively.

### Ocular surface disease index

**Fig. 5:**
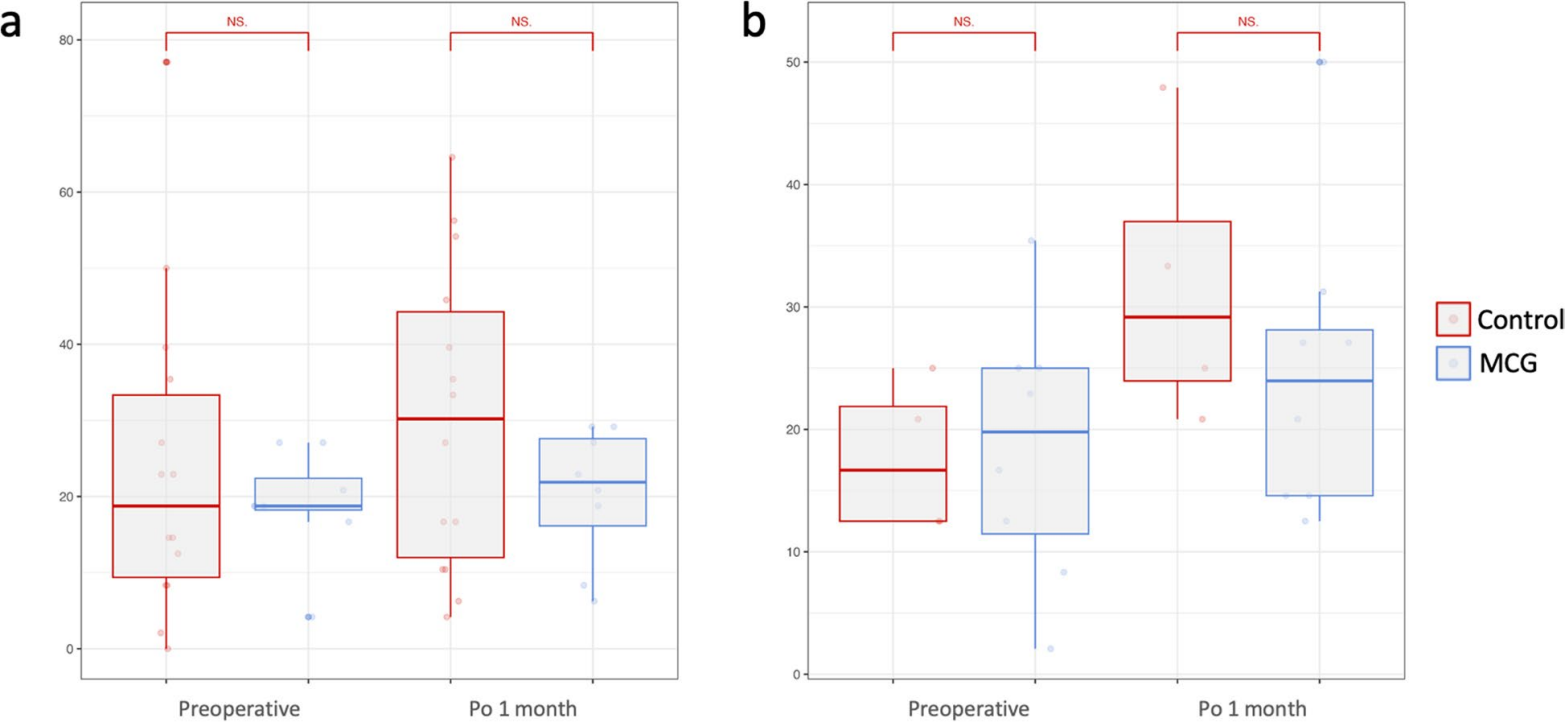
Ocular surface disease index after moisture chamber goggles treatment at postoperative 1 day, 1 week, and 1 month. **A** OSDI of control and MCG groups in SMILE participants; **B** OSDI of control and MCG groups in FS-LASIK participants. Independent student’s t-test for comparisons between control and MGC groups. OSDI, ocular surface disease index; SMILE, small-incision lenticule extraction; FS-LASIK, femtosecond laser-assisted in situ keratomileusis; PreOp, preoperative; Po, postoperative; NS, not significant

A significant increase in OSDI was observed in the FS-LASIK control group (*P* = 0.020), but not in the FS-LASIK MCG group (*P* = 0.200), or SMILE control (*P* = 0.265) and MCG (*P* = 0.663) groups (Tables 2 and 3). No statistically significant intergroup difference was observed in OSDI in either SMILE or FS-LASIK groups (Fig. 5).

## Discussion

To reduce the incidence and severity of refractive surgery-related DED, a comprehensive management approach throughout the perioperative period is needed. The current consensus recommended that both the preoperative identification and treatment of DED, and postoperative tear replacement, tear conservation, and anti-inflammation therapies, should be implemented [1, 9]. The TFOS DEWS II reports have recommended the use of MCGs for tear conservation, as they can slow tear evaporation by increasing local humidity and minimizing airflow [9]. A number of studies have investigated MCGs’ short-term efficacy in DED patients, as well as in patients exposed to adverse environments [11–13]. However, further evidence is required for the long-term efficacy of MCGs in DED patients, and their efficacy in refractive surgery-related DED. This study is the first to investigate the long-term effects of MCG on the DED parameters and visual outcomes in participants receiving refractive surgery.

Our results demonstrate that MCG treatment effectively conserved tear and stabilized the postoperative tear film. TMH was significantly higher in the MCG group compared to control 1 week and 1 month postoperatively in SMILE participants, and 1 day in FS-LASIK participants. Despite the lack of statistical significance, TMH was generally higher in the MCG groups in both SMILE and FS-LASIK participants. Previous studies reported a significant increase in TMH after the application of moisture chamber goggles [11, 12]. By maintaining a higher level of humidity inside the chambers, MCG effectively decreased the evaporation of the tear film.

In both SMILE and FS-LASIK control groups, NIBUT at postoperative 1 day postoperative decreased significantly compared to preoperatively, but not in the corresponding MCG groups. This is consistent with previous observations that MCG can elongate BUT and stabilize the tear film [12]. In the FS-LASIK control group, the decrease in NIBUT persisted 1 month after surgery, possibly due to the more significant corneal subbasal nerve dysfunction and ocular surface inflammation [3, 19]. However, this effect was reversed by MCG treatment, and a longer NIBUT was maintained in the FS-LASIK MCG group.

MCG can ameliorate conjunctival congestion after refractive surgery. The congestion scores were generally lower in the MCG groups compared to the control groups. The effect of MCG on the lipid layer of the tear film is less clear. Average LLT was significantly lower in the FS-LASIK MCG group 1 week postoperatively. No significant trend in the homogeneity of lipid layer distribution was observed. As MCG mainly inhibit the evaporation of the tear film, they are less likely to affect the lipid layer. A previous study on MCG with heating functions showed that increased temperature can lead to increased LLT [11]. Further studies are required to clarify the effects of MCG on the lipid contents in the tear film.

A significant decrease in total HOA was observed after both SMILE and FS-LASIK surgery and further improved by the use of the MCG. In the FS-LASIK MCG group, total HOA was significantly lower compared to the control group, at 1 day and 1 week after surgery. Previous studies have demonstrated that DED was associated with increased HOAs and poor visual quality and that DED interventions have led to improvement in both tear film stability and HOAs [20, 21]. This is consistent with our findings that improvements in NIBUT, TMH, and HOAs were observed in tandem after treatment with MCG.

The postoperative OSDI scores were generally lower in the MCG groups compared to the control, despite the lack of statistical significance. This demonstrates that the use of MCG can ameliorate postoperative discomfort in both SMILE and FS-LASIK recipients. In addition, a significant increase in OSDI scores was observed in the FS-LASIK control group, reflecting a higher level of discomfort induced by this surgery. This observation was consistent with previous reports that SMILE was associated with less postoperative discomfort than FS-LASIK [3, 5].

This is the first study investigating the efficacy of MCG on DED after refractive surgery. Previous studies mainly focused on the short term effects of MCG, making this study an important addition to current knowledge on the effects of moisture chamber goggles. This study uses non-invasive techniques for tear film evaluations, thereby reducing the disturbance of tear film during examinations. This study has a few important limitations. The variance in NIBUT and LLT is large, despite repeated examinations. However, this variation was consistent with previous reports [18]. Our study was conducted in the span of a year. The changes in temperature and humidity might have contributed to the variation in the results. However, the DED evaluations were conducted in an air-conditioned room, reducing the variations in temperature and humidity. In addition, potential bias might originated from differences in participants’ life styles, such as screen usage, near-work activities, cosmetics, and nutrition, etc.[22]. Future studies of DED should take lifestyle variations into consideration, and investigate potential interactions between lifestyle and the disease. Another limitation is the small sample size. However, the sample size in our study was comparable with previous reports evaluating the effects of MCG [11–13]. Future research with a larger cohort and a better-controlled environment is required to further evaluate the efficacy of MCG.

In conclusion, our results demonstrated the efficacy of MCG in refractive surgery-related DED. MCG usage effectively slowed tear evaporation, increased tear film stability, improved HOAs, and potentially reduced postoperative inflammation and discomfort in patients receiving SMILE and LASIK surgeries. MCG is a promising adjuvant therapy in the comprehensive management of refractive surgery-related DED.

## Data Availability

The datasets supporting the conclusions of this article are available upon request to the corresponding author.

## Abbreviations

DED: Dry eye disease
MGC: Moisture chamber goggle
SMILE: Small-incision lenticule extraction
FS-LASIK: Femtosecond laser-assisted in situ keratomileusis
HOA: Higher-order aberration
NIBUT: Non-invasive tear film break-up time
TMH: Tear meniscus height
LLT: Lipid layer thickness
OSDI: Ocular surface disease index
TFOS: Tear Film and Ocular Surface
DEWS: Dry Eye Workshop
PUMCH: Peking Union Medical College Hospital
SE: Spherical equivalent
ACD: Anterior chamber depth
LogMAR: Logarithm of the minimal angle of resolution
BCVA: Best corrected visual acuity
UCVA: Uncorrected visual acuity
IOP: Intraocular pressure
ICU: Interferometric color unit
AvgICU: Average lipid layer thickness in interferometric color unit
MaxICU: Maximal lipid layer thickness in interferometric color unit
MinICU: Minimal lipid layer thickness in interferometric color unit
DevICU: Deviation in lipid layer thickness in interferometric color unit
SD: Standard deviation
IQR: Interquartile range
